# Theoretical Comparison of Optical Properties of Near-Infrared Colloidal Plasmonic Nanoparticles

**DOI:** 10.1038/srep34189

**Published:** 2016-09-26

**Authors:** Kai Liu, Xiaozheng Xue, Edward P. Furlani

**Affiliations:** 1 Dept. of Electrical Engineering, University at Buffalo SUNY, NY 14260 USA; 2 Dept. of Chemical and Biological Engineering, University at Buffalo SUNY, NY 14260 USA

## Abstract

We study optical properties of near-infrared absorbing colloidal plasmonic nanostructures that are of interest for biomedical theranostic applications: SiO_2_@Au core-shell particles, Au nanocages and Au nanorods. Full-wave field analysis is used to compare the absorption spectra and field enhancement of these structures as a function of their dimensions and orientation with respect to the incident field polarization. Absorption cross-sections of structures with the same volume and LSPR wavelength are compared to quantify differential performance for imaging, sensing and photothermal applications. The analysis shows that while the LSPR of each structure can be tuned to the NIR, particles with a high degree of rotational symmetry, i.e. the SiO_2_@Au and nanocage particles, provide superior performance for photothermal applications because their absorption is less sensitive to their orientation, which is random in colloidal applications. The analysis also demonstrates that Au nanocages are advantaged with respect to other structures for imaging, sensing and drug delivery applications as they support abundant E field hot spots along their surface and within their open interior. The modeling approach presented here broadly applies to dilute colloidal plasmonic nanomaterials of arbitrary shapes, sizes and material constituents and is well suited for the rational design of novel plasmon-assisted theranostic applications.

The interest in colloidal plasmonic nanoparticles has grown steadily in recent years as advances in particle synthesis have enabled a proliferation of applications in fields such as nanophotonics, biomedicine and analytical chemistry^1^,^2^. Many applications exploit the unique and highly tunable behavior of the particles, most notably, those that are associated with the effects of localized surface plasmon resonance (LSPR). At plasmonic resonance, there is intense absorption and scattering of incident light and highly localized field enhancement. Moreover, the LSPR wavelengths of a particle are highly dependent on its size, structure and material as well as the optical properties of the surrounding medium. The LSPR wavelength can be tuned within the ultraviolet (UV) to near-infrared (NIR) spectrum by manipulating these factors. A desired LSPR wavelength can be obtained, in principle, by controlling the dimensions and morphology of the particles during synthesis. The ability to tune the LSPR and associated behavior has proven useful for a broad range of applications involving cancer therapies^3^, Raman scattering^4^, fluorescent labeling^5^, nonlinear optical imaging^6^, biosensing^7^, among others. For example, recently colloidal plasmonic nanoparticles with more complex geometries (i.e. Au nanocages and nanostars) have been synthesized with excellent controllability and have been successfully demonstrated for enhanced biomedical imaging applications^8^,^9^,^10^.

Of particular interest are two emerging biomedical applications that directly exploit plasmon-enhanced photothermal transduction namely thermally modulated drug delivery and photothermal cancer therapy. Most plasmon-based photothermal applications *in vivo* utilize Au-based nanoparticles with LSPR wavelengths in the NIR, i.e. 650–1300 nm. This is known as the near-infrared window as these are the optical wavelengths that have the deepest penetration into tissue^11^. In this work, we investigate and compare three distinct nanostructures with demonstrated efficacy for theranostic applications: core@Au-shell^12^, Au nanorod^13^, and Au nanocage structures^14^. These particles have attracted great attentions because they can be synthesized in a controllable fashion using bottom-up chemical methods, which enables tuning of their optical properties. However, they also have drawbacks. Core-shell particles with an Au shell can have limited absorption in the NIR due to a relatively thin gold shell that is required to red-shift LSPR to that range. Nanorods have a solid metallic mass, but their absorption is a strong function of their orientation relative to the incident polarization, which results in less efficient heating for randomly oriented colloidal particles. Gold nanoframes are emerging as an alternative NIR nanomaterial for photothermal therapy^15^ and drug delivery^16^. However, their NIR optical behavior (e.g., sensitivity of LSPR to spatial orientation with respect to the incident polarization) is not obvious and needs to be determined using complex 3D computational modeling. Although the optical properties of various NIR plasmonic nanomaterials, such as Au spheres, nanorods, nanotori and nanoframes, have been reported, e.g. our previous work on photothermal-induced nanobubble generation^17^,^18^, these previous studies do not provide a systematic comparison of relevant optical properties that is needed to determine the optimal choice of NIR nanoparticles for photothermal applications, which is the focus of the present study. In summary, despite the growing interest and application of colloidal plasmonic particles for theranostics, rational design in this field is lacking and can be achieved using numerical multiphysics modelling.

## Results

**Figure 1:**
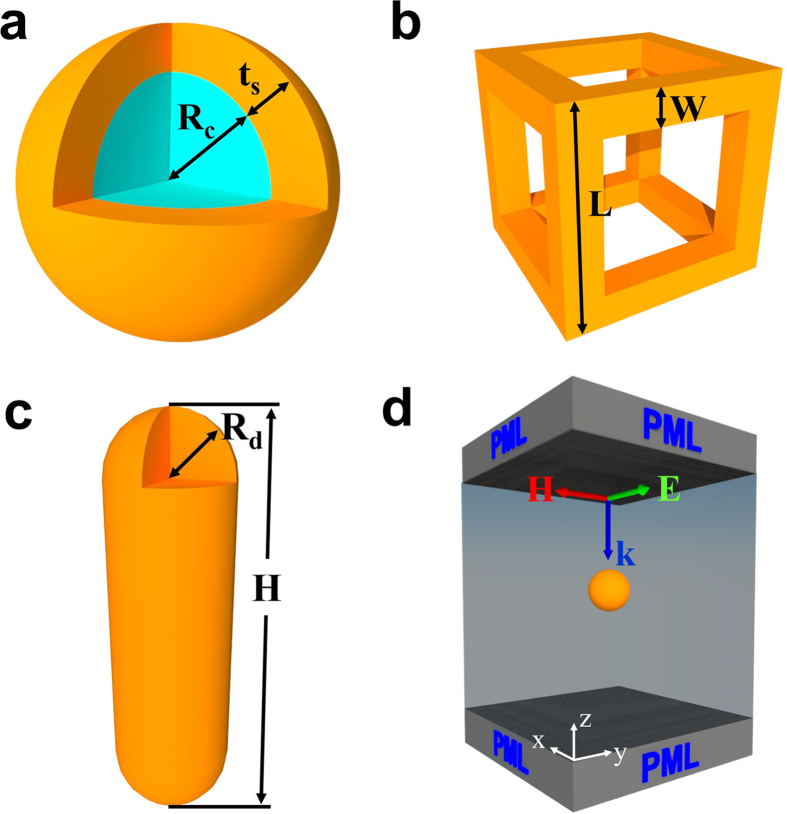
Plasmonic nanostructures and the computational model. (**a**) SiO_2_@Au core-shell particles, (**b**) Au nanocages, (**c**) Au nanorods. (**d**) Computational domain showing the polarization and propagation direction of the incident field.

We used 3D full-wave computational models to study the NIR plasmonic behavior of the three nanostructures shown in Fig. 1. In our analysis, we place more emphasis on optical absorption rather than scattering as we are interested in photothermal applications in which the absorption is the dominant factor that determines the efficiency of the system. In addition, we consider subwavelength nanoparticles for which absorption dominates scattering. The comparison between intensities of absorption and scattering can be found in the Supplementary Information. The core-shell particles consist of a silica (SiO_2_) core with a radius *R*_*c*_ and a gold shell with a thickness *t*_*s*_ as shown in Fig. 1a. The Au nanocages are cubic with twelve frame elements in the form of square Au nanowires, as shown in Fig. 1b. The nanocage geometry is defined by its length *L*, which defines the size of the cube, the width *W* that defines the cross-sectional area of the nanowire, and the aspect ratio *R* = *L*/*W*. In the literature, this structure is also referred to a nanoframe^14^. The nanorod geometry, shown in Fig. 1c, consists of a cylindrical body of radius *R*_*d*_ with hemispherical dome-shaped caps at either end. The total length of the nanorod is *H*. An example of the computational domain for this analysis is shown in Fig. 1d. Here, a single core-shell particle is centered at the origin of the domain and immersed in a carrier fluid, which we take to be H_2_O. The computational model is described in detail in the Method section.

### LSPR vs. Particle Dimensions

**Figure 2:**
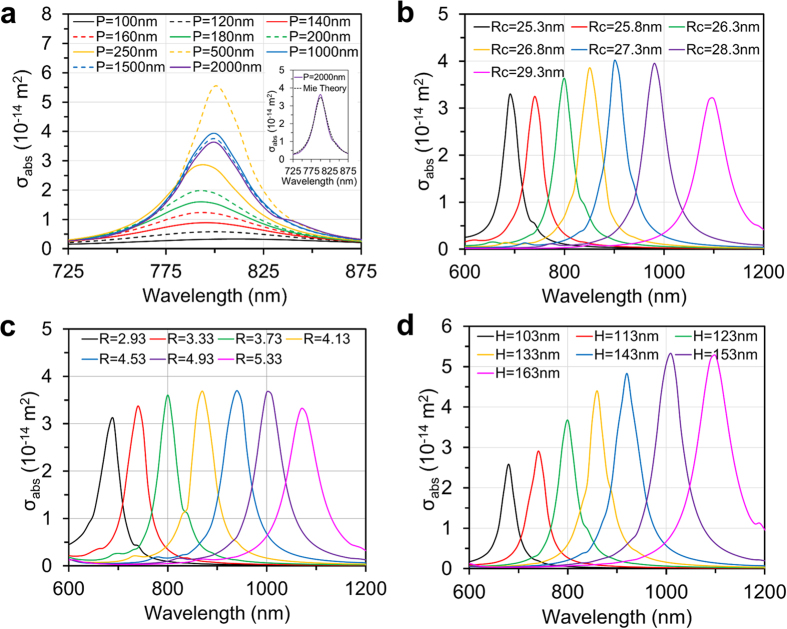
Absorption cross section spectra *σ*_*abs*_ of particles vs. variation of the domain period and dimensions. (**a**) *σ*_*abs*_ of the SiO_2_@Au particle with *R*_*c*_ = 27.3 nm and *t*_*s*_ = 3.7 nm vs. variation of the domain period *P*. The inset shows the results predicted by Mie theory. (**b**–**d**) *σ*_*abs*_ of three NIR colloids with the same volume *V*_*p*_ vs. variation of dimensions: (**b**) SiO_2_@Au core-shell particles, (**c**) Au nanoframes and (**d**) Au nanorods.

We first study the LSPR tunability of the three nanostructures as a function of their dimensional parameters. The total particle volume is held fixed at *V*_*p*_ = (50 nm)^3^ for all particles. It is important to note that the fixed particle volume applies throughout this work and hence the volume fractions of the different colloids are identical. We also assume that the colloids are sufficiently dilute so that interparticle photonic coupling is negligible. We begin by investigating the LSPR tunability of the SiO_2_@Au structure. We calibrate and validate the 3D computational model for this structure using Mie theory. To this end, Fig. 2a shows an analysis of the absorption spectrum of a SiO_2_@Au particle as a function of the size of the computational domain (Fig. 1d). Here, the length *P* that defines the square cross section of the computational domain, i.e. traverse to the direction of propagation, is systematically increased until the computed absorption spectrum equals that obtained using Mie theory. This occurs when *P* = 2000 nm as seen in the inset of Fig. 2a. This value of *P* is used throughout this work unless specified otherwise. This preliminary calibration is necessary because symmetry boundary conditions (BCs) are imposed on the lateral sides of the computational domain (i.e. transverse to the direction of propagation) to simplify the analysis. However, these BCs give rise to undesired interparticle coupling, which can contribute to the field solution and needs to be minimized by choosing a sufficiently large spacing between the particles (i.e. sufficiently large *P*) as described in the Method section.

Once the computational model is calibrated for the SiO_2_@Au particle, we study LSPR tunability wherein the SiO_2_ core is enlarged and the shell thickness is reduced, i.e. *R*_*c*_ is systematically increased from 25.3 nm to 29.3 nm (*t*_*s*_ decreases from 5.7 nm to 1.7 nm). This produces a corresponding shift in the LSPR wavelength in the NIR from 690 nm to 1100 nm, as shown in Fig. 2b. A similar analysis was performed for the nanocage. In this case, *L* is fixed at 50 nm and the aspect ratio *R* = *L*/*W* is increased from 2.93 to 5.33 (i.e. *W* decreases from 17 nm to 9.38 nm), which produces a redshift in the LSPR wavelength from 690 nm to 1070 nm as shown in Fig. 2c. Lastly, for the Au nanorods, both the length *H* and radius *R*_*d*_ need to change in order to maintain a constant volume. As *H* increases from 103 nm to 163 nm (*R*_*d*_ decreases from 21.16 nm to 16.17 nm) and the LSPR peak red-shifts from 680 nm to 1110 nm as shown in Fig. 2d.

This analysis shows that the three nanostructures are comparable with respect to their use for a prescribed NIR operating wavelength as the LSPR of each particle can be tuned to the NIR by controlling their dimensions during synthesis. This NIR tunability is especially attractive for laser-based theranostic applications as discussed above. In the remaining sections, we perform an optical analysis of three specific structures that have a LSPR wavelength of 800 nm: a SiO_2_@Au core-shell particle with *R*_*c*_ = 27.3 nm and *t*_*s*_ = 3.7 nm, a nanocage with *L* = 50 nm and *W* = 13.4 nm and a nanorod with *H* = 123 nm and *R*_*d*_ = 19 nm.

### LSPR vs. Spatial Orientation

**Figure 3:**
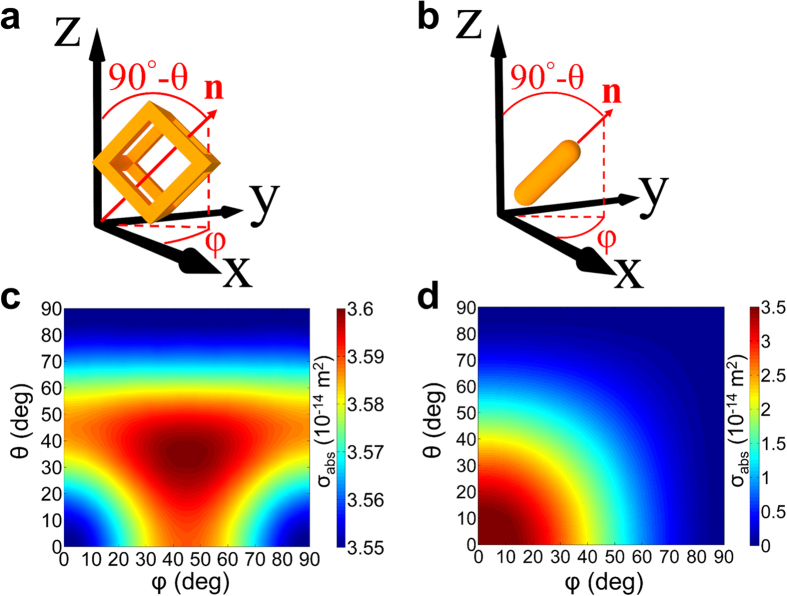
NIR absorption cross section vs. particle orientation. Geometry and coordinates of spatial orientation of (**a**) nanoframe and (**b**) nanorod; *σ*_*abs*_ vs. spatial orientation (*φ, θ*) for (**c**) Au nanoframe and (**d**) Au nanorod.

Next, we study the absorption of the nanostructures as a function of their orientation relative to the incident polarization. This is important because colloidal particles have random orientations that can impact their absorption. The SiO_2_@Au particles are centrosymmetric and therefore their absorption cross section is independent of orientation. However, nanocages and especially nanorods, have less rotational symmetry and their absorption changes with orientation. We use angles *φ* and *θ* to define the rotation of the particles relative to the x- and z-axis, respectively, as shown in Fig. 3. In Fig. 3a,b, the unit vector **n** is shown that is normal to the top area of the nanocage and along the principle axis of the nanorod, respectively. The angle *φ* lies in the x-y plane and is measured from the x-axis to the projection of **n** onto x-y plane, whereas 90° − *θ* is the angle between **n** and the z-axis.

We begin with an analysis of the nanocage as defined in the previous section (*L* = 50 nm and *W* = 13.4 nm) and compute *σ*_*abs*_ as a function of its orientation. The incident field is fixed at the LSPR wavelength of 800 nm, which is located within the NIR biological window and aligned with one of the most popularly used laser lines, i.e. 808 nm. As shown in Fig. 3c, there is very little variation in *σ*_*abs*_ throughout the entire range of orientations. This is in sharp contrast to the Au nanorod (*H* = 123 nm and *R*_*d*_ = 19 nm), which exhibits a strong orientation dependent absorption as shown in Fig. 3d. Specifically, the amplitude of *σ*_*abs*_ decreases from its maximum to zero as the nanorod rotates away from its alignment with the field polarized along its long axis (*φ* = 0°, *θ* = 0°). This orientation dependence of absorption can limit the use of nanorods for applications.

### Field Enhancement Analysis

Another important feature of the plasmonic particles is their ability to generate highly localized enhanced fields at resonance. Such “hot spots” have been exploited for many applications, including fluorescent-based imaging, controlled drug delivery, nonlinear optics, surface-enhanced Raman scattering (SERS), and various biosensing modalities. In this section, we compare the field enhancement for particles that have the same volume and LSPR wavelength, i.e. the SiO_2_@Au core-shell particle with *R*_*c*_ = 27.3 nm and *t*_*s*_ = 3.7 nm, and the nanocage with *L* = 50 nm and *W* = 13.4 nm. Since the Au nanorod is not an optimal choice for colloidal applications due to its undesirable sensitivity to its spatial orientation, its spatial profiles of local field enhancement is only briefly discussed in the Supplementary Information. Recall that these particles have comparable LSPR absorption cross-sections: *σ*_*abs*_ = 3.63 × 10^−14^ m^2^ and 3.59 × 10^−14^ m^2^, respectively.

**Figure 4:**
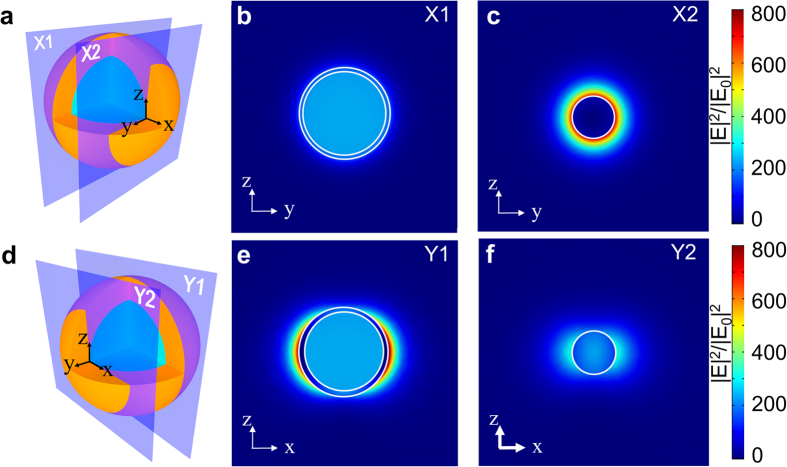
Local field enhancement of the SiO_2_@Au core-shell particle (*R*_*c*_ = 27.3 nm and *t*_*s*_ = 3.7 nm) at the LSPR wavelength of 800 nm. (**a**,**d**) Illustrate four designated planes. (**b**,**c**,**e**,**f**) plot the profiles of LSPR-induced local field enhancement. The incidence is polarized along x direction.

In Fig. 4, we plot the spatial profile of E field intensity enhancement (|***E***|^2^/|***E***_***0***_|^2^) for the SiO_2_@Au core-shell particle across four different cut planes. As shown in Fig. 4a, two yz-planes (perpendicular to the field polarization) are labelled X1 and X2 where X1 is a symmetry plane through the center of the particle and X2 is parallel to X1 and tangential to the SiO_2_ core. Figure 4b shows a weakly confined mode within the Au shell in X1 and Fig. 4c shows a relatively strong mode distributed around the outer surface of Au shell in X2. The latter is due to the resonant dipolar moment of the Au core-shell.

Figure 4d illustrates two xz-planes (parallel to the polarization), which are denoted Y1 and Y2, where Y1 cuts across the center of particle and Y2 is tangential to the SiO_2_ core. Since Y1 is aligned with the polarization, the LSPR dipolar resonance gives rise to a strongly concentrated E field with an enhancement factor over 800 as depicted in Fig. 4e. In contrast, the local field profile in Fig. 4f also shows a similar dipolar resonance mode, however the field intensity is much weaker, which is mainly due to the proximity of Y2-plane to the pole in y direction.

**Figure 5:**
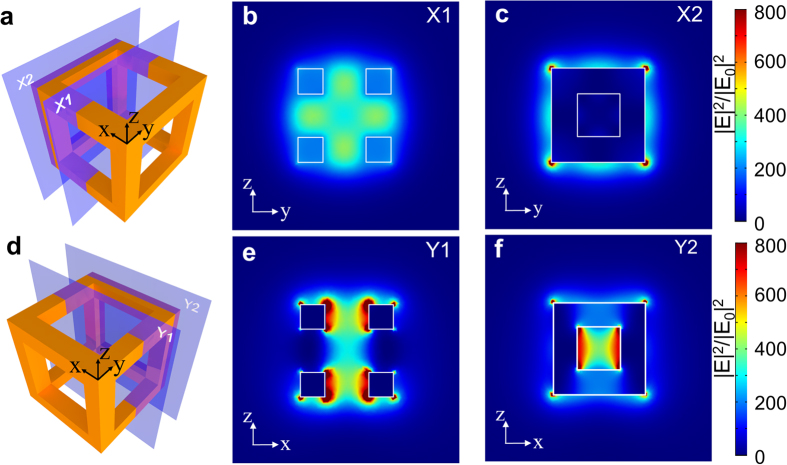
Local field enhancement profiles of the Au nanocage (*L* = 50 nm and *W* = 13.4 nm) at the LSPR wavelength of 800 nm. (**a**,**d**) Are the conceptual schematics showing four designated planes. (**b**,**c**,**e**,**f**) show the profiles of LSPR-induced local field enhancement. The incidence is polarized along x direction.

The E field enhancement profiles for the Au nanocage are shown in Fig. 5. Four planes are chosen to render the field plots. As shown in Fig. 5a, two planes X1 and X2 are perpendicular to the polarization direction (x-axis). X1 overlaps the central symmetry plane and X2 cuts the middle of the nanowires that form the edge of the structure. Figure 5b shows uniformly enhanced field intensity in X1 across the hollow interior of the nanocage. This region can potentially be loaded with theranostic agents that can be modulated by the enhanced field. Figure 5c illustrates several strongly enhanced hot spots at the outer surface of edge nanowires, which are primarily due to the dipolar resonance in those nanowires as they are aligned parallel to the polarization. Two additional planes Y1 and Y2 are defined perpendicular to the y-axis as illustrated in Fig. 5d. Strong field enhancement profiles can be observed in the hollow interior of the Au nanocage in Fig. 5e,f. This localized field concentration in the interior of the nanocage is attributed to the strong mode coupling between adjacent Au nanowire frame elements^19^,^20^. The unique advantage of the nanocage over the core-shell particle is the abundance of coupled modes existing among the Au nanowires. The nanocage provides a larger number of hot spots on its surface that can be leveraged for theranostic applications. Moreover, the surface of nanocage can be functionalized with biotargeting agents to enable selective binding to a target biomaterial, e.g. cancer cells. Specifically, by manipulating thiolate-Au monolayer chemistry, excellent compatibility between Au surfaces and various molecules and ligands can be achieved^21^,^22^. During the functionalization process, fluorescent labels can be attached to the Au surface to enable spatial tracking and imaging^15^. The LSPR of the nanoparticles can be used to enhance fluorescent signal intensity^23^, i.e. to dramatically increase the signal intensity from surface-bound or encapsulated fluorescent molecules^24^,^25^. The enhanced fluorescence could enable high-resolution *in vivo* spatial imaging and tracking.

## Discussion

Colloidal nanoparticles with tunable plasmonic behavior are increasingly used to enable new, and enhance exsiting, theranostic applications, e.g. imaging, sensing, photothermal hyperthermia, thermally-induced therapeutic nanobubble generation and optically activated drug delivery with controlled release. Many such applications require operation within the NIR biological window. Accordingly, we have used 3D computational models to compare the NIR optical behavior of three plasmonic nanoparticles with demonstrated biomedical efficacy: SiO_2_@Au core-shell, Au nanocage and Au nanorod nanostructures. We have found that while the LSPR of each of these structures can be readily tuned to the NIR biological window, there are significant differences in their behavior that impact their selection for a given application. Specifically, our analysis demonstrates the advantages of the core-shell and nanocage structures over the nanorods in terms of the absorption cross-section, insensitivity to spatial orientation and local field enhancement. In general, particles with a higher degree of rotational symmetry provide more efficient photothermal transduction because their absorption cross-section is less sensitive to their orientation, which is random in a colloid. However, while the optical absorption efficiencies of the SiO_2_@Au and nanocage structures are comparable, the latter has more abundant E-field hot spots along its surface and especially within its open interior that can be leveraged for imaging, plasmon enhanced sensing and optically controlled drug release. Lastly, the computational approach applied here provides insight into fundamental mechanisms that govern the plasmonic behaviors of colloidal nanoparticles. It is useful for the rational design of plasmonic nanoparticles for a wide range of applications.

## Methods

As noted above, we used 3D full-wave computational models to study the NIR plasmonic behaviors of the nanostructures (Fig. 1a–c). Specifically, we used the finite element (FE)-based Radio Frequency (RF) solver module in the commercial COMSOL multiphysics program (COMSOL Version 5.2, www.comsol.com). A typical computational domain (CD) is shown in Fig. 1d. Here, a single core-shell particle is centered at the origin of the domain and immersed in H_2_O. The particle is illuminated with a uniform downward-directed plane wave with the E field polarized along the x-axis. The height of the CD is 1000 nm and perfectly matched layers (PMLs) (200 nm in height) are applied at the top and bottom of the domain to reduce backscatter from these boundaries. Perfect electric conductor (PEC) conditions are applied at the boundaries perpendicular to E, and perfect magnetic conductor (PMC) conditions are applied at the boundaries perpendicular to H. It is important to note that these symmetry BCs mimic the response of an infinite 2D array of coplanar identical nanoparticles with a center-to-center *x* and *y* lattice spacing equal to the spatial period *P* = *P*_*x*_ = *P*_*y*_ of the CD. Thus, the field solution within the CD contains contributions from particles that exist outside the CD. The magnitude of these contributions, and hence their significance, depends on the lattice spacing, i.e. the spatial period of the CD. In our preliminary analysis below, we used Mie theory to calibrate the computational domain, i.e. to determine value of *P* that is large enough so that the field contributions from particles outside the CD are negligible, i.e. so that the analysis accurately reflects the optical response of a single isolated colloidal particle^26^.

We use full-wave time harmonic field theory for the analysis. As shown in Fig. 1d, an incident plane wave is launched from the top surface of the domain and propagates downward towards the bottom PML. A source current is used to generate the field as described in the literature^27^,^28^,^29^,^30^. The incident light is p-polarized at normal incidence with the E field along the x-axis and the H field along the y-axis. The time-harmonic E field within the domain satisfies the equation:


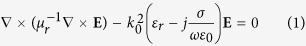


where *μ*_*r*_, *ε*_*r*_ and *σ* are the relative permeability, permittivity and conductivity of the media, respectively. In the computational model, we compute the power absorbed by the particle *Q*_*abs*_ (W) and then use this to compute the cross section *σ*_*abs*_ = *Q*_*abs*_/*I*_*laser*_, where *I*_*laser*_ (W/m^2^) is the incident irradiance. To model the nanoparticles, we need expressions for optical constants ε_r_ = (*n* − i*k*)^2^ of Au (*ε*_*Au*_), SiO_2_ (*n*_*SiO2*_) and the background medium (*n*_*H2O*_). Moreover, we need to consider the fact that the metallic materials (e.g. Au shell) can be thinner than the mean free path of free electrons (~42 nm). A dielectric function for gold that accounts for electron-surface scattering is expressed in equation 2^31^.


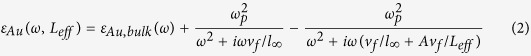


where *ε*_*Au*,*bulk*_ is the bulk dielectric function of gold, 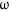 is the angular frequency of incident light, *ω*_*p*_ = 0.93 eV is the plasma frequency, *v*_*f*_ = 1.4 × 10^15^ nm/s is the Fermi velocity, *l*_∞_ = 42 nm is the mean free path of the free electrons, *A* is a dimensionless parameter, usually assumed to be close to unity (*A* = 1) and *L*_*eff*_ = *t*_*s*_ is the reduced effective mean free path of the free electrons. The bulk dielectric function is given by an analytical expression equation 3 that is based on an experiment-fitted critical points model^32^,^33^,^34^. The detailed descriptions of parameters in equation 3 can be found in the literatures^32^,^33^,^34^. The material SiO_2_ is assumed to be lossless, i.e. *k*_*SiO2*_ = 0, with a dispersive index of refraction defined in equation 4^31^. The refractive index of the nonabsorbing water surrounding is expressed in equation 5^31^. Moreover, all materials in our model have the permeability of *μ*_*r*_ = 1.


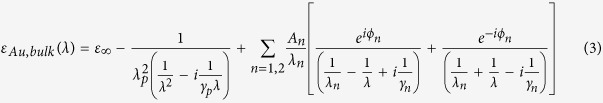



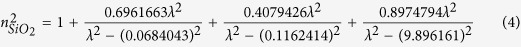



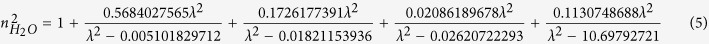


## Additional Information

**How to cite this article**: Liu, K. *et al*. Theoretical Comparison of Optical Properties of Near-Infrared Colloidal Plasmonic Nanoparticles. *Sci. Rep.* **6**, 34189; doi: 10.1038/srep34189 (2016).

## Supplementary Material

Supplementary Information
