# Supplementary material for: Theoretical Comparison of Optical Properties of Near-Infrared Colloidal Plasmonic Nanoparticles
Source: Sci Rep. 2016 Sep 26;6:34189. doi: 10.1038/srep34189 (PMC5035923; doi:10.1038/srep34189)
Supplement: Supplementary Information [file srep34189-s1.pdf]

Supplementary Information

Theoretical Comparison of Optical Properties of Near-Infrared Colloidal Plasmonic Nanoparticles

Kai Liu,[1] Xiaozheng Xue,[2] and Edward P. Furlani,[1,2,*]

[1]Dept. of Electrical Engineering, University at Buffalo SUNY, NY 14260

[2]Dept. of Chemical and Biological Engineering, University at Buffalo SUNY, NY 14260.

[*]E-mail : efurlani@buffalo.edu

Comparison of Absorption and Scattering Spectra

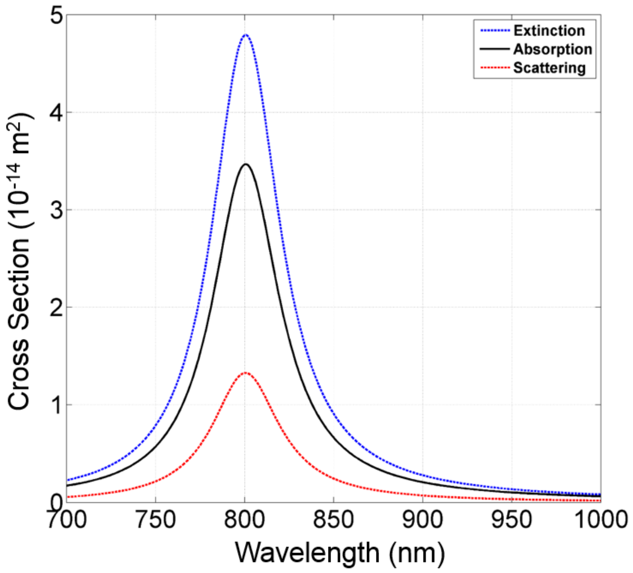

Figure S1. Comparison of Absorption and Scattering Cross Section Spectra of the SiO_2@Au core-shell particle (R_c=27.3 nm and t_s=3.7 nm): absorption (σ_{abs}), scattering (σ_{scat}) and extinction (σ_{ext}) cross sections are calculated based on Mie theory to demonstrate the less significance of σ_{scat} compared with the amplitude of σ_{abs}.

The data presented in **Fig. S1** is calculated based on Mie theory. The peaks of absorption and scattering cross sections (i.e. σ_{abs} and σ_{scat}) are aligned at the same LSPR wavelength of 800 nm. However, the amplitude of σ_{abs} is 2.6-fold as large as σ_{scat}. This significant difference is primarily attributed to the fact that the optical absorption is more dominant over the scattering in relatively

small plasmonic nanoparticles. The results justify a weaker emphasis on the analysis of scattering spectra in our current study.

Field Enhancement in Au Nanorod

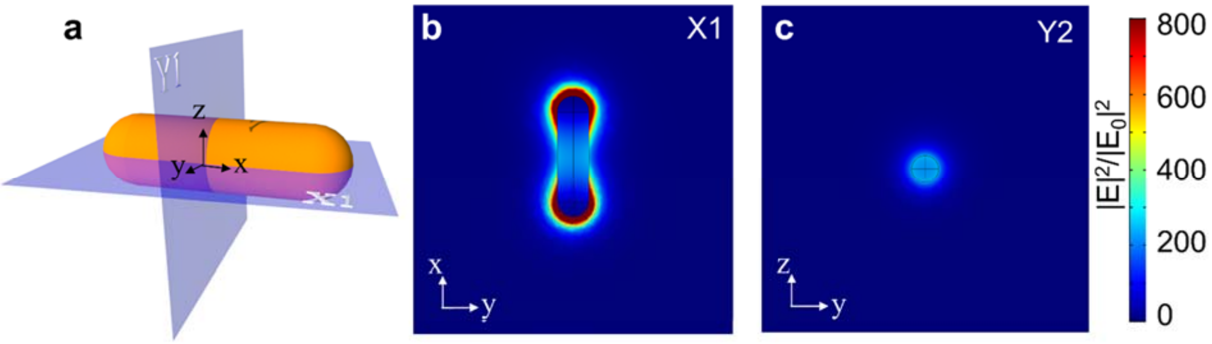

Figure S2. Local field enhancement of the Au nanorod (*H*=123 nm and R_d=19 nm) at the LSPR wavelength of 800 nm: (**a**) illustrate two designated planes X1 and Y1. (**b**)-(**c**) plot the profiles of LSPR-induced local field enhancement. The incidence is polarized along x direction.

Fig. S2a shows two designated planes X1 and Y1 for plotting the spatial profiles of local field enhancement. The plane X1 is along the direction of the incident polarization, while Y1 is perpendicular to the polarization direction. As shown in **Fig. S2b**, the electric field is strongly concentrated at two opposite ends of the nanorod. This observation is due to the alignment between the long axis of the nanorod and the polarization direction. On the other hand, **Fig. S2c** reveals that the distribution of the electric hot spots is not uniform over the surface of the nanorod. Local fields are barely enhanced at the center of the nanorod.
